# ISG20 Restricts *BK Polyomavirus* Infection and Engages in Reciprocal Regulation with Viral Large T Antigen

**DOI:** 10.3390/microorganisms13112540

**Published:** 2025-11-06

**Authors:** Yumin Hou, Chunlan Hu, Yejing Shi, Xiaohui Zhou, Tongyu Zhu, Nannan Wu

**Affiliations:** 1 Shanghai Public Health Clinical Center, Fudan University, Shanghai 201508, China; houyumin@shphc.org.cn (Y.H.); lan5969@163.com (C.H.); shiyejing@shphc.org.cn (Y.S.); zhouxiaohui@shphc.org.cn (X.Z.); 2 Department of Kidney Transplantation, Zhongshan Hospital, Fudan University, Shanghai 200032, China

**Keywords:** interferon-stimulated gene 20, *BK polyomavirus*, large T antigen, host-virus interaction

## Abstract

*BK polyomavirus* (BKPyV) causes severe urinary tract diseases, including BKPyV-associated nephropathy (BKPyVN) and ureteric stenosis, in immunocompromised individuals such as renal transplant recipients. Effective antiviral therapies for BKPyV infection remain an unmet clinical need. While the interferon-stimulated gene 20 (ISG20) exhibits broad-spectrum antiviral activity against RNA viruses, its role and mechanisms against DNA viruses are poorly defined. This study demonstrates, for the first time, potent antiviral activity of ISG20 against BKPyV. This restriction was observed with both endogenous levels of ISG20 and upon overexpression, and this effect was confirmed by ISG20 knockout and immunofluorescence imaging. We observed that ISG20 expression is dynamically regulated during BKPyV infection: it is upregulated both during early infection and by expression of the viral large T antigen (LT) alone. However, endogenous ISG20 expression becomes significantly suppressed during later stages of infection, coinciding with declining LT levels. The physical interaction between LT and both wild-type and mutant ISG20 suggests a potential viral strategy to sequester this restriction factor. These findings establish ISG20 as a novel host restriction factor against BKPyV and suggest that BKPyV employs LT-mediated mechanisms to evade or counteract ISG20’s antiviral effects. Our results elucidate a complex biphasic interplay between BKPyV and host innate immunity, identifying ISG20 as a potential therapeutic target for BKPyV-associated diseases.

## 1. Introduction

*BK polyomavirus* (BKPyV), a member of the polyomavirus family, exhibits seroprevalence rates exceeding 90% in adults [1]. Among kidney transplant recipients, up to 30% develop BKPyV DNAemia, with roughly a quarter of these cases progressing to *BK polyomavirus*-associated nephropathy (BKPyVN) [2]. It is also associated with other severe urinary disorders such as hemorrhagic cystitis, ureteral stricture, and bladder cancer [3,4]. At present, there is no effective antiviral drug to deal with BKPyV reactivation except to reduce the intensity of immunosuppression, which will undoubtedly increase the risk of organ rejection [2]. Therefore, the development of novel anti-BKPyV drug targets is urgent.

BKPyV is a typical double-stranded DNA virus, whose genome encodes early regulatory proteins (large T antigen, LT; and small T antigen, sT) and late structural proteins (VP1, VP2, VP3, and Agno), initiated by bidirectional promoters, respectively [5]. Once entering the cell, BKPyV uses cellular replication, transcription and translation machineries to synthesize its own DNA, mRNA and proteins, which increases the difficulty of specific antiviral treatment [6,7]. While the innate immune response to BKPyV in its natural target cells is critically important, it remains poorly characterized. Previous research indicated that BKPyV infection failed to activate innate immune pathways in the renal tubular epithelial cells. Conversely, BKPyV infection was shown to activate the IFN-β-dependent Type I interferon pathway in pulmonary vascular endothelial cells, leading to the upregulation of interferon-stimulated genes (ISGs), including interferon-stimulated gene 20 (ISG20) [8].

ISG20 is an interferon-stimulated protein belonging to the DEDDh exonuclease family, exhibiting both RNase and DNase activity. To date, antiviral research on ISG20 has primarily focused on RNA viruses, with no reported inhibitory effect on common DNA viruses such as adenovirus [9]. Point mutations in the conserved nuclease domain of ISG20 (D94G) result in a loss of RNase activity and a concurrent loss of antiviral activity, supporting the prevailing view that ISG20 exerts its antiviral effects through viral RNA cleavage [10]. While ISG20 is established as an anti-RNA virus effector, emerging evidence suggests its antiviral portfolio may be broader. Notably, ISG20 has also been shown to target the RNA transcripts of the DNA virus Hepatitis B Virus (HBV), thereby inhibiting its replication [11].However, our previous study demonstrated that ISG20 plays an antiviral role by inhibiting the translation of viral proteins [12], which echoes the findings of some studies regarding the inhibitory effect of ISG20 on Venezuelan equine encephalitis virus (VEEV), Chikungunya virus (CHIKV), and hepatitis C virus (HCV) [13,14].

Beyond its antiviral roles, ISG20 is implicated in renal pathophysiology. It is upregulated in clear cell renal cell carcinoma and may serve as a potential biomarker [15]. Furthermore, elevated ISG20 expression in the kidney following ischemia-reperfusion injury reduces cellular stress and helps prevent further damage to renal cells [16,17]. Despite its documented significance in kidney diseases, the role of ISG20 in the context of BKPyV infection remains unexplored.

We previously established a BKPyV infection system in TCCSUP cells, a urothelial-derived line that is relevant to the primary site of BKPyV replication [18], and observed that infection induced endogenous ISG20 expression. In this study, we demonstrate that ISG20 suppresses BKPyV infection at both endogenous and overexpression levels, likely through a post-transcriptional mechanism. Interestingly, while ISG20 expression was upregulated during the early phase of BKPyV infection, it was subsequently downregulated, suggesting a dynamic virus-host interplay. Further investigations are needed to elucidate the exact mechanism by which ISG20 interacts with BKPyV.

## 2. Materials and Methods

### 2.1. Cells

Human bladder carcinoma TCCSUP cell line (ATCC, #HTB-5, Manassas, VA, USA) was cultured in MEM/EBSS medium (Cytiva, #SH30024.01, Marlborough, MA, USA). Human embryonic kidney 293T (ATCC, #CRL-11268) and 293TT cell lines (ATCC, #CRL-3467), as well as African green monkey kidney Vero cells (ATCC, #CCL-81), were maintained in DMEM (Cytiva, #SH30243.01). Human Primary Renal Proximal Tubule Epithelial Cells (hRPTECs; ATCC, #PCS-400-010) were cultured in renal epithelial cell basal medium (ATCC, #PCS-400-030) supplemented with the renal epithelial cell growth kit (ATCC, #PCS-400-040). All cells were supplemented with 10% fetal bovine serum (Lonsera, #S711-001S, Shanghai, China), 100 U/mL penicillin and 100 μg/mL streptomycin (Gibco, #15140122, Waltham, MA, USA), and cultured in a humidified atmosphere with 5% CO_2_ at 37 °C.

### 2.2. Plasmids

N-terminal Flag-tagged human ISG20 and its nuclease-deficient mutant D94A (ISG20M1) were cloned into pcDNA3.1 (Invitrogen, Waltham, MA, USA) as described in [12]. BKPyV-UT strain (ATCC, #VR-837) was used as a template for large T antigen (forward 5′-GCCACCATGGATAAAGTTCTTAACAGGG-3′ and reverse 5′-TTATTTTGGGGGTGGTG-3′) and cloned into pcDNA3 through the *Eco*RI and *Xho*I restriction sites. The remaining plasmids were obtained from Addgene (Cambridge, MA, USA): LentiCRISPR v2 (#52961), psPAX2 (#12260), pMD2.G (#12259), lentiCRISPRv2 neo (#98292), pwB2b (#32094, expressing BKPyV-VP1/VP2/VP3), pwB3b (#32106, expressing BKPyV-VP1/VP3), and pEGFP-N1 (#172281).

### 2.3. Stable Cells

ISG20 knockout TCCSUP cells were generated by clustered regularly interspaced short palindromic repeats (CRISPR)-associated systems (CRISPR/Cas9). Briefly, a single ISG20 sgRNA was designed to target the human ISG20 open reading frame (ORF) (5′-CATGGCTGGGAGCCGTGAGG-3′) and cloned into the lentiCRISPR v2 plasmid. To produce lentivirus, 293T cells were transfected with the lentiCRISPR v2-sgRNA-ISG20 plasmid, the envelope plasmid pMD2.G, and the packaging plasmid psPAX2 (at a ratio of 5:3:2). The culture supernatant (lentiviral suspension) was collected 72 h post-transfection and used to infect TCCSUP cells. 24 h post-infection, the medium was replaced with a selection medium containing 1.5 μg/mL puromycin (Sigma, #540411, Saint Louis, MO, USA), ultimately leading to the establishment of the ISG20-knockout (ISG20-KO) TCCSUP polyclonal bulk population cells.

ISG20-overexpressing TCCSUP cells were generated by lentivector-based retroviral gene transduction, similar to the process described above. Briefly, the human ISG20 gene with a V5 tag was synthesized (Sangon Biotech, Shanghai, China) and cloned into the modified lentiCRISPRv2 neo vector. Lentivirus was produced in 293T cells and used to infect TCCSUP cells, which were then selected with 200 μg/mL G418 (Sigma, #A1720). This process resulted in the establishment of ISG20-overexpressing (ISG20-OE) TCCSUP polyclonal bulk population cells.

### 2.4. Viruses

The BKPyV-UT strain amplified in Vero cells, and purified through density gradient ultracentrifugation as described in [19]. The titer of the newly prepared virus was determined by immunofluorescence assay in RPTEC.

Single-round BK pseudovirus (SrBKPV) was generated as detailed in [20]. Briefly, 293TT cells were co-transfected with capsid protein expression plasmids pWB2b and pWB3b, along with the reporter plasmid pEGFP-N1, 48 h post-transfection, cells were harvested and lysed in PBS (Corning, #21-040-CV, New York, NY, USA) containing 9.5 mM MgCl_2_ (Sigma-Aldrich, #M9272, Saint Louis, MO, USA), 25 mM ammonium sulfate (Sigma-Aldrich, #A4915), 0.5% (*v*/*v*) Triton X-100 (Sigma-Aldrich, #T8787), and 0.1% (*v*/*v*) RNase A/T1 cocktail (Thermo Fisher Scientific, #EN0551, Waltham, MA, USA). Following overnight incubation at 37 °C, lysates containing mature capsids were cleared by two 10 min centrifugations at 5000× *g*. Virus particles were purified via ultracentrifugation (234,000× *g*, 3.5 h) through a 25% (*w*/*v*) sucrose cushion and resuspended in 100 μL Opti-MEM medium (Gibco, #31985070). SrBKPV titer was determined by quantifying GFP-positive 293T cells using flow cytometry (BD).

### 2.5. IFN-β Antiviral Assay

In order to test the antiviral effect of IFN-β on BKPyV, TCCSUP cells were seeded in 6 cm dishes (4 × 10^5^ cells per dish) and pre-treated with IFN-β (25, 50, 100, and 200 U/mL) for 24 h. Subsequently, the cells were infected with BKPyV at an MOI of 1 in serum-free MEM for 2 h at 37 °C, and then the medium was replaced with complete culture medium for 3 d. The cells were collected for immunoblotting or qPCR.

In order to test the dose-dependent and time-dependent effects of IFN-β on the induction of ISG20 expression, TCCSUP cells were seeded in 6-well plates (2 × 10^5^ cells per well) overnight and treated with increasing doses of recombinant human IFN-β (R&D Systems, #8499-IF-010, Minneapolis, MN, USA) (10, 25, 50, 100, and 200 U/mL) for 24 h, after which the cells were collected for subsequent experiments. Similarly, cells were treated with 200 U/mL of IFN-β and harvested at designated time points (2, 4, 8, 12, 24, 48, and 72 h).

### 2.6. ISG20 Antiviral Assay

Wild-type, ISG20-OE, or ISG20-KO TCCSUP cells were seeded in 6 cm dishes (4 × 10^5^ cells per dish) overnight and infected with BKPyV-UT at MOIs of 0.1, 0.25, 0.5, 1, and 2 in serum-free MEM for 2 h. The cells were washed once with PBS, and fresh medium was added. Cells were harvested on day 3 post-infection.

For rescue assays, ISG20-KO TCCSUP cells transfected with a Flag-ISG20 plasmid for 24 h were infected with BKPyV-UT at various MOIs (0.5, 1, and 2) for 3 d, and cells were then collected for immunoblotting.

For the transient-expression antiviral assay, Flag-ISG20 and Flag-ISG20M1 were transfected into TCCSUP cells for 24 h and then infected with BKPyV at an MOI of 0.25 under serum-free conditions. The medium was then replaced with complete culture medium, and the cells were incubated for 3 d. Cells were collected for immunoblotting and immunofluorescence.

To evaluate the antiviral activity against SrBKPV, wild-type, ISG20-OE, or ISG20-KO TCCSUP cells were seeded in 48-well plates (4 × 10^4^ cells per well) overnight and then infected with SrBKPV at an MOI of 10 for 48 h. GFP-positive cell percentages were quantified by flow cytometry.

### 2.7. Transient DNA Transfection

TCCSUP cells reached approximately 80% confluence and were transfected using a plasmid to transfection reagent ratio of 1:1.5 (1 μg plasmid to 1.5 μL transfection reagent). DNA transfections were carried out by Lipofectamine 2000 (Invitrogen, #11668019) with indicated amount or ratio of plasmids. To minimize cytotoxicity, the total plasmid amount did not exceed 4 μg per 6 cm dish or 2 μg per well in a 6-well plate.

### 2.8. Western Blotting (WB)

For protein extraction, cells were lysed on ice in cell lysis buffer (Biosharp, #BL509A, Beijing, China) containing protease inhibitor cocktail (Takara, #635673, Kusatsu, Shiga, Japan) for 20 min, followed by centrifugation at 12,000 rpm for 10 min at 4 °C to remove cell debris. After that, 25 μg protein in each well was separated by 12.5% SDS–polyacrylamide gel (Epizyme, #PG113, Shanghai, China) electrophoresis, and then transferred onto polyvinylidene difluoride membranes (Millipore, #ISEQ00005, Billerica, MA, USA). Next, the membranes were blocked with tris-buffered saline (TBS) supplemented with 5% nonfat dry milk (Epizyme, #PS112) and 0.05% Tween-20 (Sigma-Aldrich, #P1379) for 1 h at room temperature (RT). Membranes were then incubated with primary antibody diluted in 5% milk TBS-Tween at 4 °C overnight. The next day, the membrane was incubated with horseradish peroxidase (HRP)-conjugated anti-mouse IgG antibody (Sigma-Aldrich, #A9044) or HRP-conjugated anti-rabbit IgG antibody (GeneTex, #GTX213110-01, Sunnyvale, CA, USA) in 5% milk TBS-Tween for 2 h at RT. Protein bands were visualized using chemiluminescent ECL substrate (Cytiva, #RPN2235, Marlborough, MA, USA) and detected with an infrared fluorescent detection system (Bio-Rad, Hercules, CA, USA). The following primary antibodies were used: ISG20 rabbit polyclonal antibody (Proteintech, #22097-1-AP, Wuhan, China), SV40 large T antigen mouse monoclonal antibody (Abcam, #PAb416, Cambridge, UK), VP1 mouse monoclonal antibody (Abnove, #MAB3204-M02, Taiwan, China), FLAG mouse monoclonal antibody (Sigma-Aldrich, #F1804), and GAPDH antibody (GeneTex, #GTX627408-01).

### 2.9. Quantitative Real-Time PCR (qPCR)

Cells were collected and total RNA was extracted using the Direct-zol RNA Miniprep Kits (ZYMO RESEARCH, #R2052, Irvine, CA, USA). 1 μg of RNA was reverse-transcribed for each sample using the PrimeScript RT reagent Kit (Takara, #RR047A). Intracellular DNA was extracted using the MiniBEST Universal Genomic DNA Extraction Kit (Takara, #9765) according to the manufacturer’s instructions. qPCRs were performed using the TB Green Premix Ex Taq (Takara, #RR420A) and specific primers were as follows (from 5′ to 3′): ISG20 (F:ATGGCTGGGAGCCGTGAGG; R:CGGTGATCTCTCCCTCAGG), LT (F:AGGCTGGATTCTGAGATAAGT; R:CCATGTCCAGAGTCTTCAGT), VP1 (F:GCATTCGATTCCGACTCACC; R:CAACATGGAGGTGATGCCG), GFP (F:GAACGGCATCAAGGTGAACT; R:TGCTCAGGTAGTGGTTGTCG), GAPDH (F:GATTCCACCCATGGCAAATTC; R:CTGGAAGATGGTGATGGGATT) on an Applied Biosystems 7500 Real Time PCR System (Thermo Fisher Scientific, Waltham, MA, USA).

### 2.10. Immunofluorescence Staining and Microscopy

TCCSUP cells grown on chamber slides (Merck, #PEZGS0816, Darmstadt, Germany) were fixed with 4% paraformaldehyde (Coolaber, #SL1830, Beijing, China) for 10 min at RT, followed by permeabilization with 0.5% Triton X-100 (Sigma-Aldrich, #T8787) for 10 min. After blocking with 2% bovine serum albumin (BSA; Sigma-Aldrich, #V900933) in PBS for 45 min, cells were incubated overnight at 4 °C with anti-T antibody (Cell Signaling Technology, #15729, Danvers, MA, USA) and anti-Flag antibody (Sigma-Aldrich, #F1804). The next day, cells were incubated for 1 h at RT (protected from light) with Alexa Fluor-conjugated secondary antibodies—either donkey anti-rabbit antibody (Invitrogen, #A32790) or donkey anti-mouse antibody (Invitrogen, #A10037). Nuclei were counterstained with DAPI (Cell Signaling Technology, #4083) for 10 min before mounting. Images were acquired using a Tissue FAXS 200 flow-type tissue cytometer (Tissue Gnostics GmbH, Vienna, Austria) and processed using ImageJ software 1.8.0.

### 2.11. Co-Immunoprecipitation (Co-IP)

TCCSUP cells were co-transfected with BKPyV-LT and Flag-ISG20 or Flag-ISG20M1 plasmids in 10 cm dish. After culturing for 48 h, the cells were washed with PBS and the proteins were obtained as described above. Then, 30 μL of the cell lysate was saved for input control, and added 40 μL of the Anti-FLAG M2 Magnetic Beads (Sigma-Aldrich, #M8823) into the rest of the lysate. The mixture was incubated for 2 h at RT with gentle mixing. The Flag fusion proteins were eluted from the magnetic beads by competition with the FLAG^®^ peptide (Sigma-Aldrich, #F4799), All protein samples were boiled for 10 min for WB.

### 2.12. Cell Viability Assay

TCCSUP cells were seeded in 96-well plates (5 × 10^3^ cells per well) overnight and infected with BKPyV at MOIs of 0.1, 0.25, 0.5, 1, and 2 in serum-free MEM for 2 h. The cells were washed once with PBS, and fresh medium was added. Each treatment condition was performed in at least six replicate wells. Cell viability was assessed on day 3 post-infection. Similarly, TCCSUP cells were infected with BKPyV at an MOI of 1 and assessed at designated time points (1, 2, 3, 4, 5, 6, and 7d post-infection).

Cell viability was determined using the Cell Counting Kit-8 (CCK-8; Meilunbio, #MA0218-5, Dalian, China). Briefly, fresh medium containing 10% CCK-8 was prepared and added as a medium exchange to each well. The plates were then incubated for an additional 1 h at 37 °C. The absorbance of each well was measured at a wavelength of 450 nm using a microplate reader (Thermo Scientific Multiskan FC, Waltham, MA, USA).

### 2.13. Statistical Analysis

All data were analyzed using GraphPad Prism 8.0 and presented as means ± SEM. Statistical significance between two groups was determined using unpaired two-tailed Student’s *t*-test, and differences among multiple groups were analyzed by one-way ANOVA. In addition, *p* < 0.05 was considered statistically significant.

## 3. Results

### 3.1. Both IFN-β and BKPyV Upregulate the Expression of ISG20

**Figure 1:**
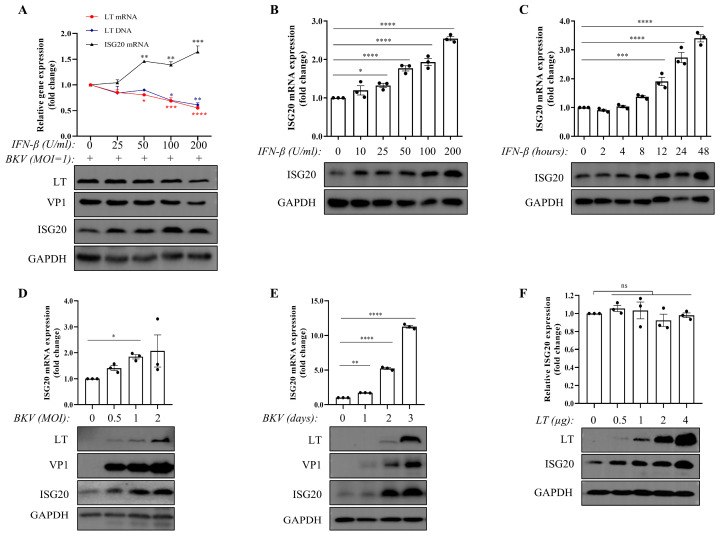
Both IFN-β and BKPyV upregulate ISG20 expression. (**A**) TCCSUP cells were pretreated with increasing doses of IFN-β (25, 50, 100, and 200 U/mL) for 24 h, then infected with BKPyV (MOI = 1) for 3 d. Viral (VP1 protein and LT levels (protein, mRNA, and DNA) and host (ISG20 protein/mRNA) markers were analyzed by WB and qPCR. Comparing all groups to the untreated control (0 U/mL IFN-β) (**B**,**C**) TCCSUP cells were treated with increasing doses of IFN-β (10, 25, 50, 100, and 200 U/mL) for 24 h (**B**), or treated with 200 U/mL IFN-β at the indicated time points (2, 4, 8, 12, 24, and 48 h) (**C**). ISG20 protein/mRNA levels were analyzed by WB and qPCR. (**D**,**E**) TCCSUP cells were infected with BKPyV at different MOIs (0, 0.5, 1, 2) for 72 h (**D**), or infected with BKPyV (MOI = 1) for 1–3 d (**E**). ISG20 protein/mRNA levels were analyzed by WB and qPCR. (**F**) TCCSUP cells were transfected with BKPyV-LT plasmid for 48 h. Cells were collected for RT-qPCR analysis of endogenous ISG20 mRNA and WB analysis of LT and endogenous ISG20 proteins. GAPDH served as a loading control. Data represent mean ± SEM (*n* = 3 replicates). The dot represents individual data points. ns: not significant, * *p* < 0.05, ** *p* < 0.01, *** *p* < 0.001 and **** *p* < 0.0001 by one-way ANOVA.

To investigate IFN-β mediated anti-BKPyV activity and the potential involvement of ISG20, TCCSUP cells were pretreated with IFN-β prior to BKPyV challenge. The results demonstrated a dose-dependent antiviral effect, characterized by reduced viral DNA, mRNA, LTand VP1 protein levels, along with increased endogenous ISG20 mRNA and protein expression (Figure 1 and Figure S1). Notably, IFN-β treatment alone directly induced ISG20 expression in TCCSUP cells in both dose- and time-dependent manners (Figure 1B,C). These findings indicate that ISG20 may be involved in the IFN-β-mediated suppression of BKPyV infection in TCCSUP cells.

Interestingly, although BKPyV infection in TCCSUP cells did not induce detectable IFN-β secretion (below the detection limit of 1.18 pg/mL), it still triggered increased ISG20 mRNA and protein expression in dose- and time-dependent manners (Figure 1D,E). These observations indicate that BKPyV may upregulate ISG20 expression through an IFN-independent pathway in TCCSUP cells. To identify the viral factor responsible for this IFN-independent ISG20 induction, we investigated the role of the major early protein, Large T antigen (LT). Strikingly, while transfection of an LT-expressing plasmid led to a clear, dose-dependent upregulation of endogenous ISG20 at the protein level, it had no effect on ISG20 mRNA levels (Figure 1F). This compelling dissociation between mRNA and protein responses demonstrates that the LT antigen is sufficient to drive ISG20 protein expression post-transcriptionally.

### 3.2. ISG20 Is an Antiviral Factor Against BKPyV

**Figure 2:**
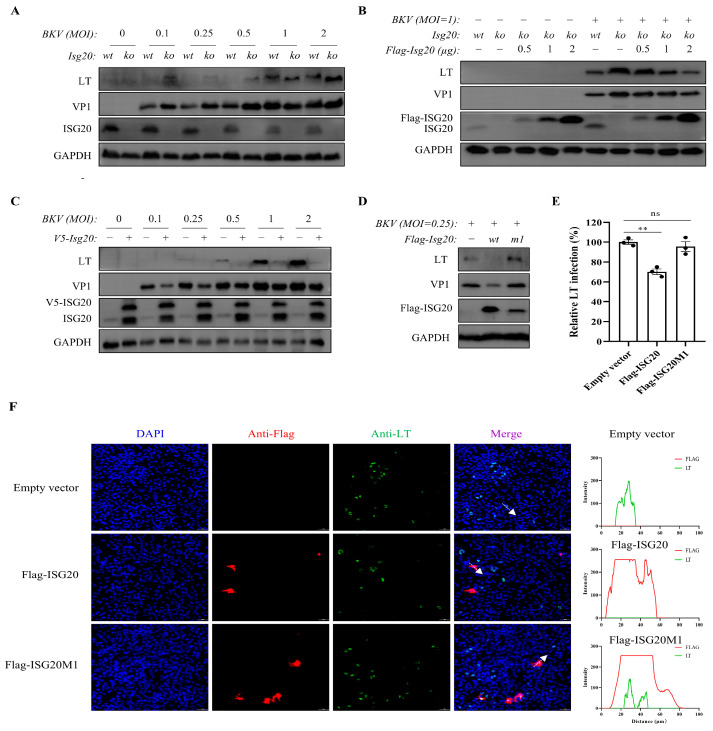
Wild-type ISG20 inhibits BKPyV, but the mutant ISG20M1 lacks this inhibitory activity. (**A**) Wild-type (WT) and ISG20-knockout (KO) TCCSUP cells were infected with BKPyV (MOI = 0.1–2) for 3 d. Cell lysates were analyzed by WB for viral (LT, VP1) and host (ISG20) protein levels. (**B**) ISG20-KO cells were transfected with increasing amounts of Flag-ISG20 plasmid (0.5–2 μg) for 24 h prior to BKPyV infection (MOI = 1). Cells were then harvested 3 d post-infection. LT, VP1 and ISG20 protein levels were assessed by WB. (**C**) WT and ISG20-overexpressing (OE) TCCSUP cells were infected with BKPyV (MOI = 0.1–2) for 3 d. Viral (LT, VP1) and ISG20 protein levels were examined by WB. (**D**) TCCSUP cells transfected with Flag-ISG20, Flag-ISG20M1, or empty vector (pCDNA3.1) for 24 h were infected with BKPyV (MOI = 0.25). LT, VP1 and Flag-tagged protein levels were analyzed by WB. (**E**,**F**) Immunofluorescence analysis was performed on transfected cells (as in (**D**)) stained with anti-Flag (red) and anti-LT (green) antibodies, with DAPI nuclear counterstain (blue). Images were acquired using Tissue FAXS 200 cytometer (Tissue Gnostics GmbH, Vienna, Austria). Scale bar: 50 μm. Immunofluorescence statistical analyses of BKPyV infection (**E**). Immunofluorescence analysis of LT and FLAG-tagged protein co-localization in mock-transfected (empty vector), Flag-ISG20-transfected, and Flag-ISG20M1-transfected TCCSUP cells. The representative immunofluorescence intensity of the arrow-indicated cell in the merged image is shown on the right line chart individually (**F**). GAPDH served as a loading control. Data represent mean ± SEM (*n* = 3 replicates). The dot represents individual data points. Statistical analyses were performed using one-way ANOVA (ns: not significant, ** *p* < 0.01).

To assess the role of endogenous and overexpressed ISG20 in BKPyV infection, we established ISG20-knockout (KO) and ISG20-overexpressing (OE) TCCSUP cell lines. ISG20 deletion enhanced viral LT and VP1 expression (Figure 2A), while transient ISG20 reintroduction restored its antiviral activity (Figure 2B). Conversely, ISG20 overexpression reduced viral protein levels (Figure 2C). These results collectively demonstrate that both endogenous and exogenous ISG20 effectively suppress BKPyV replication. To rule out the possibility that the observed antiviral effects were due to virus-induced alterations in cell viability, we infected these cell lines with BKPyV. As shown in Supplementary Figure S2, no significant loss of cell viability was observed in Wild-type, ISG20-KO, or ISG20-OE cells at 72 h post-infection compared to mock-infected controls.

To determine whether ISG20 affects viral entry, we challenged wild-type, ISG20-OE, and ISG20-KO TCCSUP cells with single-round BK pseudovirus (SrBKPV) containing full-length capsid proteins and an EGFP reporter. Flow cytometric analysis revealed comparable infection rates across all three cell types (Supplementary Figure S3), indicating that ISG20 does not significantly impair BKPyV entry.

We further investigated the functional importance of ISG20’s exonuclease domain by comparing wild-type Flag-ISG20 with its catalytic-deficient mutant (Flag-ISG20M1). Pre-transfection of wild-type ISG20, reduced LT and VP1 protein levels following BKPyV infection (Figure 2D). Notably, VP1 expression in ISG20M1-expressing cells remained comparable to vector controls. This finding was confirmed and quantified by immunofluorescence analysis, which demonstrated a significant decrease in the percentage of LT-positive cells upon expression of wild-type ISG20 (Figure 2E). Furthermore, single-cell co-expression analysis revealed a strong negative correlation between ISG20WT expression and viral antigen levels, which was absent in the catalytic mutant group (Figure 2F). Together, these data strongly support the conclusion that ISG20’s antiviral activity against BKPyV is critically dependent on its exonuclease activity.

### 3.3. Dynamic LT-ISG20 Crosstalk Balances Viral Propagation and Host Restriction During BKPyV Infection

**Figure 3:**
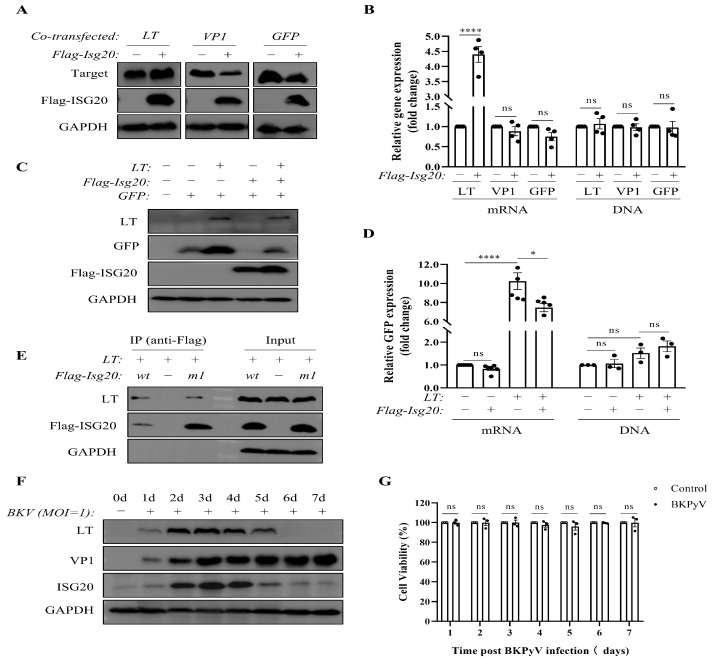
ISG20 differentially modulates protein expression, and ISG20 interacts with the LT protein. (**A**,**B**) TCCSUP cells were co-transfected with Flag-ISG20 and plasmids encoding BKPyV-LT, pwB2b, or pEGFP-N1 at a ratio of 3:1 for 48 h. Cells were collected for WB analysis of LT, VP1, GFP, and Flag-ISG20 (**A**), or for RT-qPCR and qPCR analysis of LT, VP1, and GFP mRNA and DNA levels (**B**). (**C**,**D**) TCCSUP cells were transfected or co-transfected with BKPyV-LT, Flag-ISG20, pEGFP-N1 plasmids (at a ratio of 6:6:1) for 48 h. Cells were collected for WB analysis of LT, GFP, and Flag-ISG20 (**C**), or for RT-qPCR and qPCR analysis of GFP mRNA and DNA levels (**D**). (**E**) TCCSUP cells were co-transfected with BKPyV-LT and Flag-ISG20 or Flag-ISG20M1 (at a ratio of 3:1) for 48 h. Cells were then collected for immunoprecipitation and WB analysis of LT, VP1, and Flag-tagged proteins. (**F**) TCCSUP cells were infected with BKPyV (MOI = 1) for 1–7 d. Levels of LT, ISG20, and VP1 in cell lysates were tested by WB. (**G**) TCCSUP cells were infected or not infected with BKPyV (MOI = 1) for 1–7 d. Cell viability was measured daily using the CCK-8 assay. GAPDH served as a loading control. Data represent mean ± SEM (*n* = 3 replicates). The dot represents individual data points. Statistical analyses were performed using Student’s *t*-test or one-way ANOVA (ns: not significant, * *p* < 0.05, **** *p* < 0.0001).

Previous studies have suggested that ISG20 may exert antiviral effects either through its nuclease activity or by inhibiting protein translation. To determine whether ISG20-mediated suppression of BKPyV-associated proteins occurs at the transcriptional or translational level, co-transfection experiments were performed. The results demonstrated that ISG20 exhibited translational inhibition of both the BKPyV structural protein VP1 and the positive control GFP (without affecting DNA or mRNA levels), consistent with prior findings [12]. However, ISG20 did not suppress the protein expression of the BKPyV regulatory protein LT; instead, it significantly upregulated LT mRNA and protein levels (Figure 3A,B).

Further investigation revealed that LT could upregulate both mRNA and protein expression of ISG20 and GFP, and it counteracted ISG20’s translational suppression of exogenously expressed GFP (Figure 3C,D). Co-immunoprecipitation experiments confirmed that both ISG20 and its catalytically inactive mutant ISG20M1 physically interact with LT (Figure 3E).

To further elucidate the dynamic interplay between viral proteins and ISG20 during BKPyV infection, we monitored their expression over a one-week period. VP1 levels increased progressively after infection, whereas both LT and ISG20 exhibited an initial upregulation (reaching a peak at day 3) followed by a decline (Figure 3F). Critically, we confirmed that this biphasic expression pattern was not due to virus-induced cell death, as cell viability remained high and comparable to not-infected controls throughout the 7-day course of infection (Figure 3G). These findings suggest a potential direct interaction between LT and ISG20, with LT expression levels dynamically modulating ISG20’s antiviral efficacy during BKPyV infection.

## 4. Discussion

This study provides the first evidence for the antiviral effect of the host restriction factor ISG20 against BKPyV, a representative dsDNA virus, revealing a complex viral-host arms race with three key findings: (1) ISG20 can be upregulated by both exogenous IFN-β treatment and BKPyV infection. However, the potent induction of ISG20 during infection in the absence of detectable IFN-β suggests that IFN-β-independent mechanisms likely play a major role in this process; (2) ISG20’s antiviral activity may manifest after viral entry and is most likely mediated at post-transcriptional level; (3) Overexpression of the viral large T antigen (LT) can bind to ISG20 and directly induce its expression.

ISG20 possesses both IFN-dependent and IFN-independent antiviral activities, while IFN-β exerts its broad antiviral effects by coordinating the induction of a vast network of ISGs. Therefore, although our findings position ISG20 as an important contributor within the IFN-β-mediated anti-BKPyV response, we do not exclude the significant role of other ISGs. While ISG20 is a classical interferon-stimulated gene (ISG), its induction can occur through interferon-independent pathways as part of the initial cellular intrinsic immune response [21]. Specifically, the cytosolic BKPyV DNA genome is likely sensed by the cGAS-STING axis. STING activation can directly recruit and phosphorylate transcription factors such as IRF3 and NF-κB, which are capable of binding to the promoter of ISG20 and driving its transcription without the requirement for de novo type I interferon synthesis and signaling [21,22]. This interferon-independent “first wave” of ISG induction represents a rapid frontline defense mechanism against viral infection. While the specific roles of BKPyV proteins in directly regulating ISG20 transcription remain to be fully elucidated, the large T antigen has been shown to broadly modulate host gene expression [23]. Therefore, the observed upregulation of ISG20 likely represents a component of the host’s concerted antiviral response, which the virus simultaneously attempts to subvert for its own replication. The innate immune response to BKPyV is highly cell-type-specific. While infection of vascular endothelial cells robustly activates the IFN-β pathway [8], the virus fails to activate these pathways in primary renal proximal tubular epithelial cells (RPTECs) [24], which are a major site of clinical infection and persistence.

This study demonstrates the inhibitory effect of ISG20 on BKPyV infection at both endogenous and overexpression levels. Previous research identified ISG20 as typically exhibiting antiviral activity against RNA viruses but not DNA viruses, such as adenovirus [9]. However, ISG20 displays antiviral activity against HIV and HBV, viruses whose life cycles involve both DNA and RNA stages [25,26,27], highlighting the complexity of ISG20’s antiviral activity and mechanisms. ISG20 may employ four distinct antiviral mechanisms: (1) Direct hydrolysis of viral RNA, as observed for HBV [26,27]; (2) Direct degradation of deaminated viral DNA, exemplified by its synergistic action with the deaminase APOBEC3A to inhibit HBV covalently closed circular DNA (cccDNA) levels [28]. This functional synergy between a nuclease and a deaminase is particularly intriguing in the context of polyomaviruses. Given that the *BK polyomavirus* large T antigen (LT-Ag) induces APOBEC3B expression [29], it is plausible that APOBEC3-mediated mechanisms contribute to BKPyV mutagenesis in vivo [30]. This suggests that the host employs multiple nucleic acid-editing enzymes as a coordinated defense network against viral infection.; (3) Induction of the IFN pathway and activation of interferon-induced protein with tetratricopeptide repeats 1 (IFIT1)-mediated inhibition of viral mRNA translation, as seen with chikungunya virus (CHIKV) or Venezuelan equine encephalitis virus (VEEV) [14]; (4) A fourth potential mechanism, identified in our preliminary study, involves distinguishing non-self RNA and inhibiting its translation, as observed during VSV infection where ISG20 did not activate the IFIT1 pathway yet still suppressed viral mRNA translation [12].

The mechanistic characterization of ISG20’s anti-BKPyV activity revealed several important features. The pseudovirus entry assay excluded effects of ISG20 on viral internalization. Most significantly, the catalytically inactive ISG20M1 mutant, which also lacks the ability to inhibit mRNA translation, completely lost antiviral capacity [12]. Due to technical issues and sensitivity limitations, we were unable to replicate the binding observed between LT and endogenously expressed ISG20 in the BKPyV infection system. Therefore, guided by the aforementioned research, we conducted co-transfection assays, uncovering a reciprocal regulation between LT and ISG20, which may represent a major advance in understanding BKPyV-host dynamics. While ISG20 translationally suppressed VP1 and GFP, it paradoxically enhanced LT expression—a phenomenon potentially exploitable by the virus. Remarkably, LT not only neutralized ISG20’s translational inhibition but also induced expression of co-transfected ISG20 and GFP. This result aligns with previous research indicating that LT expression from Simian Virus 40 (SV40), BKPyV, or JC virus (JCV) upregulates ISGs in mouse embryonic fibroblasts (MEFs), thereby inducing an antiviral state [31], but differs from SV40 LT’s reported inhibition of STING signaling [32]. BKPyV utilizes several immune evasion strategies, such as antigenic variation, latency, and the modulation of T-cell responses [33]. However, our data also demonstrate a significant downregulation of ISG20 during prolonged BKPyV infection, potentially reflecting a viral evasion strategy, as continuous high expression of ISG20 can be harmful to cell survival [12]. This aligns with a common viral paradigm where pathogens directly counteract host restriction factors to facilitate replication [34], for instance, the HIV-1 Vif protein targets APOBEC3G for degradation [35], and the Influenza A virus NS1 protein binds to and inhibits PKR [36]. The physical interaction between LT and both wild-type and mutant ISG20 suggests a potential viral strategy to sequester this restriction factor. The synchronous dynamic expression of LT and ISG20 during infection may reflect an evolutionary balance: early LT-mediated ISG20 induction could prevent excessive viral spread, while later-phase decline may facilitate viral persistence.

Therapeutically, disrupting this specific virus-host interface presents an attractive strategy. Currently, there are no clinically approved drugs that directly target ISG20 or its interaction with viral proteins. However, our discovery that the catalytically inactive ISG20M1 mutant still interacts with LT opens a promising avenue. It suggests that small molecules or peptide mimetics could be designed to disrupt the LT-ISG20 physical interaction. Such compounds would aim to liberate ISG20, allowing it to exert its full antiviral potential without necessarily affecting its enzymatic function or host cell physiology. While developing such targeted therapies faces significant challenges, including achieving specificity and efficient delivery, it represents a novel approach distinct from conventional antivirals and merits future investigation.

## 5. Conclusions

Our work in the human bladder carcinoma TCCSUP cell line delineates a complex equilibrium between BKPyV and host defenses, where ISG20 serves as both a viral restriction factor and interacts with the viral large T antigen. Whether this dynamic interplay influences viral pathogenesis and persistence, such as in BKPyVN, remains to be determined. However, our findings offer a novel mechanistic basis for such investigations and highlight ISG20 as a potential therapeutic target.

## Data Availability

The original contributions presented in this study are included in the article/Supplementary Material. Further inquiries can be directed to the corresponding authors.

## Supplementary Materials

The following supporting information can be downloaded at: https://www.mdpi.com/article/10.3390/microorganisms13112540/s1, Figure S1. IFN-β pretreatment dose-dependently inhibited BKPyV. TCCSUP cells were pretreated with increasing doses of IFN-β (25, 50, 100, and 200 U/mL) for 24 h, then infected with BKPyV (MOI=1) for 3 d. VP1 mRNA and DNA were analyzed by qPCR. Comparing all groups to the untreated control (0 U/mL IFN-β). Data represent mean ± SEM (*n* = 3 replicates). * *p* < 0.05, ** *p* < 0.01, *** *p* < 0.001 and **** *p* < 0.0001 by one-way ANOVA. Figure S2. BKPyV infection has no effect on the viability of TCCSUP cells. WT, ISG20-KO, and ISG20-OE TCCSUP cells were infected with BKPyV at MOIs of 0.1, 0.25, 0.5, 1, 2, or left uninfected for 3 d. Cell viability was measured by CCK-8 assay. Data represent mean ± SEM (*n* = 3 replicates). The dot represents individual data points. Statistical analyses were performed using one-way ANOVA (ns: not significant). Figure S3. ISG20 has no effect on BKPyV entry into TCCSUP cells. WT, ISG20-KO, and ISG20-OE TCCSUP cells were infected with SrBKPV at an MOI of 10 for 48 h. The percentage of GFP-positive cells was measured by flow cytometry, and statistical analysis of the infection is presented. Data represent mean ± SEM (*n* = 3 replicates). The dot represents individual data points. Statistical analyses were performed using one-way ANOVA (ns: not significant).

## Abbreviations

The following abbreviations are used in this manuscript:

BKPyV	*BK polyomavirus*
WB	Western blot
qPCR	Quantitative Polymerase Chain Reaction
MOI	Multiplicity of Infection
GAPDH	Glyceraldehyde3-phosphate dehydrogenase
ECL	Enhanced Chemiluminescence
ISG	Interferon-Stimulated Gene
IFN	Interferon
CO-IP	Co-immunoprecipitation
MEM/EBSS	Minimum Essential Medium with Earle’s Balanced Salt Solution
DMEM	Dulbecco’s modified Eagle’s medium
EGFP	Enhanced Green Fluorescent Protein
DAPI	4′,6-Diamidino-2-Phenylindole
SEM	Standard Error of the Mean
CCK-8	Cell Counting Kit-8
