# Supplementary material for: ISG20 Restricts BK Polyomavirus Infection and Engages in Reciprocal Regulation with Viral Large T Antigen
Source: Microorganisms. 2025 Nov 6;13(11):2540. doi: 10.3390/microorganisms13112540 (PMC12654336; doi:10.3390/microorganisms13112540)
Supplement: Supplementary file 1 [file microorganisms-13-02540-s001.zip › microorganisms-3807144-supplementary.pdf]

Supplementary Figures.

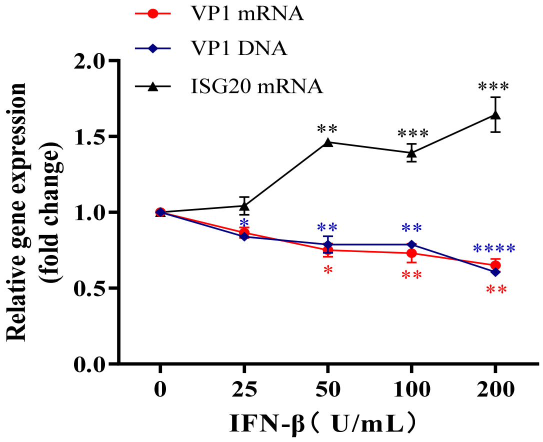

Figure S1. IFN-β pretreatment dose-dependently inhibited BKPyV. TCCSUP cells were pretreated with increasing doses of IFN-β (25, 50, 100, and 200 U/mL) for 24 h, then infected with BKPyV (MOI=1) for 3 d. VP1 mRNA and DNA were analyzed by qPCR. Comparing all groups to the untreated control (0 U/mL IFN-β). Data represent mean ± SEM (n=3 replicates). * $p < 0.05$, ** $p < 0.01$, *** $p < 0.001$ and **** $p < 0.0001$ by one-way ANOVA.

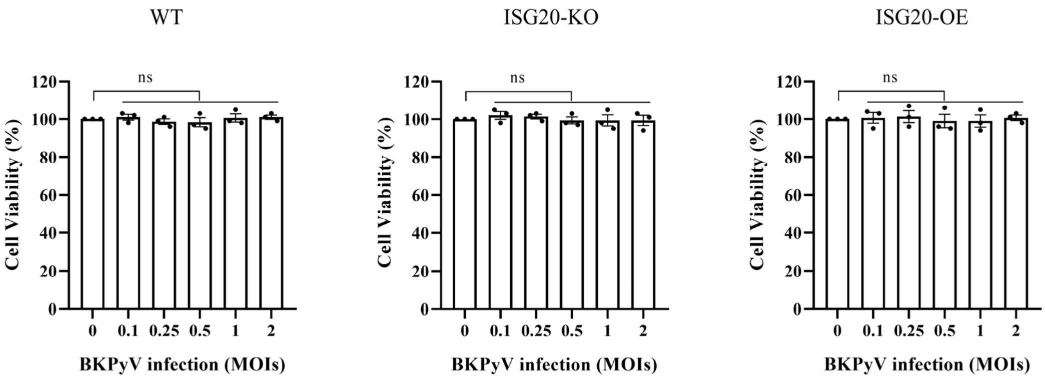

Figure S2. BKPyV infection has no effect on the viability of TCCSUP cells. WT, ISG20-KO, and ISG20-OE TCCSUP cells were infected with BKPyV at MOIs of 0.1, 0.25, 0.5, 1, 2, or left uninfected for 3 d. Cell viability was measured by CCK-8 assay. Data represent mean ± SEM (n = 3 replicates). The dot represents individual data points. Statistical analyses were performed using one-way ANOVA (ns: not significant).

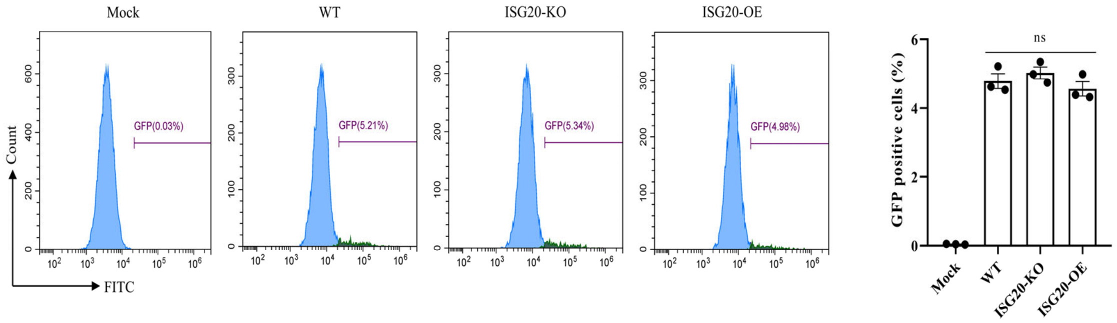

Figure S3. ISG20 has no effect on BKPyV entry into TCCSUP cells. WT, ISG20-KO, and ISG20-OE TCCSUP cells were infected with SrBKPV at an MOI of 10 for 48 h. The percentage of GFP-positive cells was measured by flow cytometry, and statistical analysis of the infection is presented. Data represent mean ± SEM (n = 3 replicates). The dot represents individual data points. Statistical analyses were performed using one-way ANOVA (ns: not significant).
